# Working memory and pattern separation in founder strains of the BXD recombinant inbred mouse panel

**DOI:** 10.1038/s41598-021-03850-3

**Published:** 2022-01-07

**Authors:** Price E. Dickson, Guy Mittleman

**Affiliations:** 1 Department of Biomedical Sciences, Joan C. Edwards School of Medicine, Marshall University, 1700 3rd Ave., Huntington, WV 25703 USA; 2 Department of Psychological Science, Ball State University, North Quad (NQ), Room 104, Muncie, IN 47306 USA; 3 Department of Psychology, University of Memphis, 400 Innovation Drive, Memphis, TN 38111 USA

**Keywords:** Genetics, Behavioural genetics, Operant learning, Short-term memory

## Abstract

Working memory and pattern separation are fundamental cognitive abilities which, when impaired, significantly diminish quality of life. Discovering genetic mechanisms underlying innate and disease-induced variation in these cognitive abilities is a critical step towards treatments for common and devastating neurodegenerative conditions such as Alzheimer's disease. In this regard, the trial-unique nonmatching-to-location assay (TUNL) is a touchscreen operant conditioning procedure allowing simultaneous quantification of working memory and pattern separation in mice and rats. In the present study, we used the TUNL assay to quantify these cognitive abilities in C57BL/6J and DBA/2J mice. These strains are the founders of the BXD recombinant inbred mouse panel which enables discovery of genetic mechanisms underlying phenotypic variation. TUNL testing revealed that pattern separation was significantly influenced by mouse strain, whereas working memory was not. Moreover, horizontal distance and vertical distance between choice-phase stimuli had dissociable effects on TUNL performance. These findings provide novel data on mouse strain differences in pattern separation and support previous findings of equivalent working memory performance in C57BL/6J and DBA/2J mice. Although working memory of the BXD founder strains was equivalent in this study, working memory of BXD strains may be divergent because of transgressive segregation. Collectively, data presented here indicate that pattern separation is heritable in the mouse and that the BXD panel can be used to identify mechanisms underlying variation in pattern separation.

## Introduction

Working memory and pattern separation are fundamental cognitive abilities which, when impaired, significantly diminish quality of life. Consequently, substantial effort in the neurosciences has been directed towards discovering biological mechanisms underlying variation in these cognitive abilities^1–15^. Nevertheless, we are only beginning to understand the genetic mechanisms that underlie variation in working memory and pattern separation^16–19^. Discovering and characterizing these mechanisms is a critical step towards treatments for common and devastating psychiatric disorders such as Alzheimer's disease. Because the mouse is an essential genetics tool for accomplishing these goals^20,21^, developing and optimizing mouse behavioral assays that can precisely detect differences in working memory and pattern separation is a critical step towards achieving this goal.

**Figure 1:**
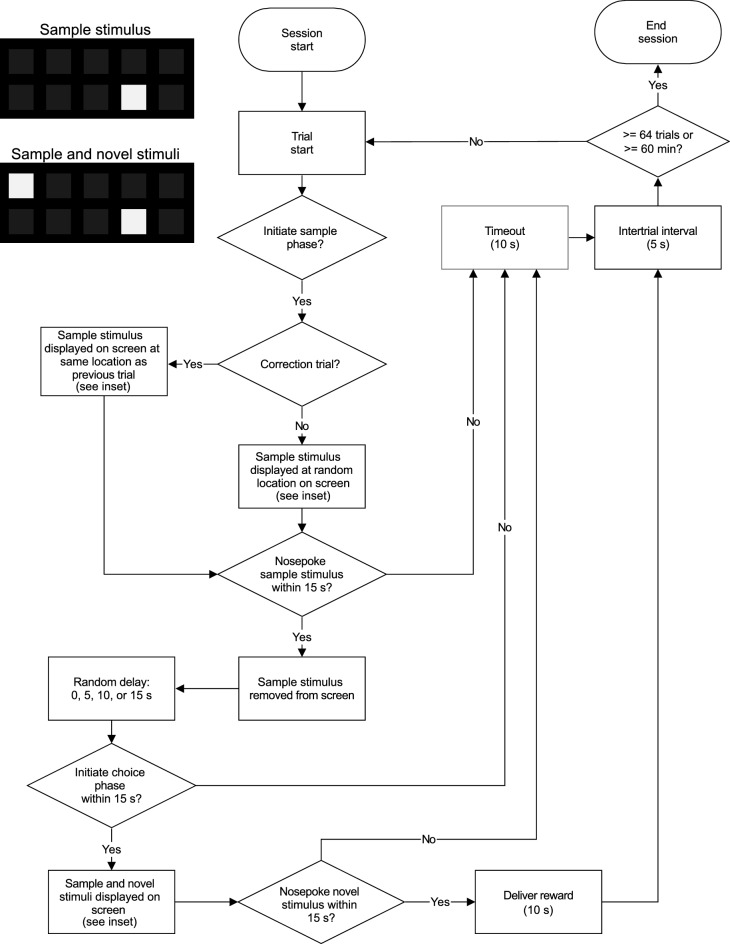
The TUNL assay for quantification of working memory and pattern separation in C57BL/6J and DBA/2J mice. The flowchart illustrates the different phases of the TUNL assay. The inset illustrates the stimuli that were displayed on the touchscreen during the sample phase and the choice phase. Dark gray stimuli shown in the inset illustrate the matrix of possible locations for the sample and novel stimuli; in the TUNL experiment, only the white sample stimulus and white novel stimulus were shown on the touchscreen. During correction trials, both the sample stimulus and novel stimulus were displayed at the same locations as they were displayed in the preceding trial. During non-correction trials, the sample stimulus and novel stimulus were displayed at random locations.

The trial-unique delayed nonmatching-to-location assay (TUNL) is a relatively new operant conditioning procedure initially developed for rats which simultaneously indexes working memory and pattern separation^22,23^. The TUNL assay consists of two phases: a sample phase during which the subject is presented with a location that must be remembered and a choice phase during which memory for that location is quantified. During the sample phase, the subject is presented with a white stimulus on a black background displayed on a touchscreen at a randomly presented position in a matrix of rows and columns (Fig. 1 inset, top). The subject must nosepoke the stimulus after which it disappears from the screen. A brief delay of variable duration follows, typically on the scale of seconds, during which the subject must hold in working memory the location of the sample. The choice phase follows this delay. During the choice phase, the mouse is presented with two white stimuli on the touchscreen: the previously presented sample stimulus and a novel stimulus presented at a randomly selected position in the matrix of possible stimulus positions (Fig. 1 inset, bottom). The subject must nosepoke the novel stimulus (i.e., the one that was not presented during the sample phase) to receive a food reward. On each trial, the length of the delay is experimentally manipulated to probe working memory, and the distance between the sample stimulus and novel stimulus is experimentally manipulated to probe pattern separation. The TUNL assay was developed to improve on the delayed non-matching to position assay by (1) adding a pattern separation component, (2) allowing the location of the sample stimulus and novel stimulus to vary independently across trials, and (3) reducing the ability of the subject to use mediating strategies to choose the correct stimulus. To date, no studies have used the TUNL assay to quantify differences in working memory and pattern separation between mouse strains. Establishing the existence of mouse strain differences on the TUNL assay is an important goal because it would establish the magnitude of working memory and pattern separation heritability in mice. Moreover, establishing strain differences would be the first step towards using a systems genetics approach to identify genetic mechanisms underlying these fundamental cognitive abilities.

In the present study, we used the TUNL assay to quantify working memory and pattern separation in the C57BL/6J and DBA/2J inbred mouse strains. We chose these strains because they are the two founder strains of the BXD recombinant inbred mouse panel^21^. The BXD panel can be used in the context of a systems genetics approach to discover genetic mechanisms underlying phenotypic variation^24–31^. Confirmation of strain differences in the BXD founders on the TUNL assay would provide evidence of the viability of this approach for identifying genetic mechanisms driving working memory and pattern separation variation. C57BL/6J and DBA/2J mice acquired the TUNL assay using the widest horizontal distance between the two stimuli during the choice phase and in the absence of a delay between the sample phase and the choice phase. Once mice had acquired this simplified version of the TUNL assay, we probed working memory by introducing a delay between the sample phase and choice phase which varied across trials (0, 5, 10, or 15 s); we probed pattern separation by varying horizontal distance and vertical distance between the sample stimulus and novel stimulus. These variables were randomized independently on each trial. Using these data, we assessed the effects of mouse strain, delay between the sample phase and choice phase, horizontal distance between choice-phase stimuli, vertical distance between choice-phase stimuli, and interactions among these variables on the TUNL assay.

## Materials and methods

### Subjects and housing conditions

Experiments were conducted in The Department of Psychology at The University of Memphis and approved by the Institutional Animal Care and Use Committee at the University of Memphis. Experiments were conducted in accordance with the National Institutes of Health Guidelines for the Care and Use of Laboratory Animals and with the ARRIVE guidelines. Efforts were made to reduce the number of animals used and to minimize animal pain and discomfort.

Male C57BL/6J mice (n = 22; JAX stock number 000664) and male DBA/2J mice (n = 21; JAX stock number 000671) were weaned at 4 weeks of age and were used as experimental subjects. These relatively large group sizes were chosen to ensure sufficient statistical power. After weaning at 4 weeks of age, experimental subjects were housed in groups of 3–5 in standard shoebox cages until they entered the experiment at 10–18 weeks of age, at which point they were individually housed. Mice had free access to water at all times. Mice had free access to food until they were individually housed, at which point they were food restricted to 90% of baseline weight. Mice were maintained in a temperature-controlled environment (21 ± 1 °C) on a 12:12 light:dark cycle (lights on at 0800).

### Apparatus

Training and testing were conducted in eight operant conditioning chambers which have been described in detail previously^32^. Briefly, the front wall of each chamber consisted of an infrared touchscreen. The rear wall consisted of (1) a centrally mounted liquid dipper which provided access to 0.01 cc of Silk Vanilla Soymilk as a reward, (2) a stimulus light located above the food receptacle, and (3) a house light centrally mounted at the top of the chamber. Operant conditioning chambers were controlled by a Lafayette Instruments control unit running ABET II and Whisker software. All operant conditioning schedules were written in-house using ABET II.

### Operant conditioning

#### Pretraining and training for the TUNL assay

The pretraining stage used for the TUNL assay was similar to the one we have previously used for touchscreen reversal learning and attentional set shifting^32–35^. Briefly, mice learned to use the touchscreen operant conditioning chamber over several pretraining stages (Table S1). Once fully pretrained, mice could complete the following sequence of behaviours: (1) nosepoke the liquid dipper receptacle to initiate the sample phase, (2) nosepoke a visual stimulus presented on the touchscreen, (3) nosepoke the liquid dipper receptacle to initiate the choice phase, (4) nosepoke a visual stimulus presented on the touchscreen, (5) collect a food reward delivered via the liquid dipper.

**Figure 2:**
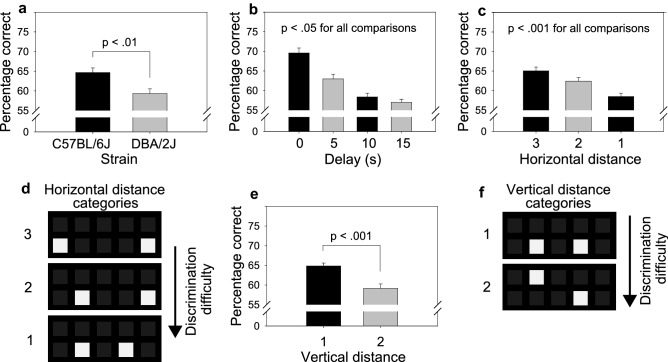
TUNL performance was significantly influenced by strain, delay, horizontal distance, and vertical distance in C57BL/6J and DBA/2J mice. (**a**) TUNL performance of C57BL/6J mice was significantly better than performance of DBA/2J mice. **(b)** Increasing the delay between the sample phase and choice phase significantly impaired TUNL performance. **(c,d)** Increasing the horizontal distance between choice-phase stimuli significantly *facilitated* TUNL performance. **(e,f)** In contrast, increasing the vertical distance between choice-phase stimuli significantly *impaired* TUNL performance. Error bars represent SEM.

TUNL training began as a simplified version of the TUNL testing stage described in the next section. Specifically, during TUNL training, delays between the sample phase and choice phase as well as horizontal distance between choice-phase stimuli (Fig. 2d) were slowly introduced across nine training stages (Table S2). Both categories of vertical distance (Fig. 2f) were used during all stages of TUNL training. On each TUNL training stage, mice were required to reach a criterion of 80% correct on trials that used a 0 s delay and the widest horizontal distance between choice-phase stimuli (i.e., the least challenging discrimination) before moving to the next TUNL training stage. Once mice had reached that criterion on each of the nine training stages, TUNL testing began.

#### TUNL testing

During TUNL testing (Fig. 1), each trial began with the illumination of the house light and the stimulus light. When mice nosepoked the food receptacle which was located directly below the stimulus light, the stimulus light was extinguished and the sample stimulus was randomly presented at one of the ten locations in the 5 × 2 matrix on the touchscreen (Fig. 1 inset, top). When mice nosepoked the stimulus, it was immediately removed from the screen. Following this, one of four delays (0, 5, 10, 15 s) occurred. During the delay, the house light was on, the rear stimulus light was off, and nothing was displayed on the touchscreen. The delay was randomly selected on each trial. At the end of the delay, the rear stimulus light was illuminated to indicate that the choice phase could be initiated by a nosepoke to the food receptacle. When mice nosepoked the food receptacle, the stimulus light was extinguished and the choice phase began. During the choice phase, two stimuli were simultaneously presented on the screen (Fig. 1 inset, bottom). The first was the sample stimulus (the incorrect choice) which was presented in the same location as it was presented during the sample phase. The second was the novel stimulus (the correct choice) which was presented at a random location in the 5 × 2 matrix on the touchscreen. The horizontal distance (Fig. 2d) and vertical distance (Fig. 2f) between the sample stimulus and the novel stimulus varied randomly and independently on each trial. If mice nosepoked the correct stimulus during the choice phase, a reward was delivered by raising the liquid dipper for 10 s. If mice nosepoked the incorrect stimulus during the choice phase, a timeout was delivered by extinguishing the house light for 10 s. A five second inter-trial interval followed the reward or timeout. The next trial began following the inter-trial interval.

As is typically done in mouse operant conditioning assays which use a forced choice component^22,32–39^, correction trials were used in the TUNL assay. Specifically, if an incorrect choice was made on the choice phase of the trial, the same pattern of stimuli was presented on the next trial. This continued until the mouse made a correct choice on the choice phase. This strategy was employed to avoid the mouse developing an intractable side bias in which all choices were to the right or left side. Performance on correction trials was not considered in the analysis of performance on the TUNL assay.

To ensure that mice were “on task” during TUNL testing, 15-s intermediate holds were used following (1) presentation of the sample stimulus, (2) illumination of the stimulus light to indicate the choice phase could be initiated, and (3) presentation of the stimuli during the choice phase. Specifically, at each of these points during the trial, if mice failed to nosepoke a stimulus on the screen or initiate the choice phase by nosepoking the food receptacle within 15 s, the trial was terminated. The session was terminated when mice completed 64 trials (excluding omitted trials) or when 60 min had elapsed, whichever occurred first.

### Statistical methods

Analysis of variance (ANOVA) was used to assess the effects of the independent variables and the interactions among these variables on the dependent variables. Percentage correct was the primary dependent variable; neither omitted trials nor correction trials were included in the calculation of percentage correct. Strain (C57BL/6J, DBA/2J) was a between-subjects factor. Within-subjects factors were delay between the sample phase and choice phase (0, 5, 10, 15 s), horizontal distance between choice-phase stimuli (1, 2, 3; ordered from least distance to greatest distance between the two stimuli), and vertical distance between choice-phase stimuli (1, 2; ordered from least distance to greatest distance between the two stimuli). Henceforth, these variables are abbreviated as delay, horizontal distance, and vertical distance, respectively. The relative position of the two choice-phase stimuli in the horizontal distance and vertical distance categories is shown in Fig. 2d,f.

ANOVAs were conducted using the GLM or UNIANOVA command in SPSS. If the ANOVA contained a within-subjects factor, the F statistic, the *p* value, the factor or interaction degrees of freedom, and the error degrees of freedom were reported from the Wilks’ Lambda row of the multivariate tests table. If an ANOVA contained a between-subjects factor, these values were reported from the between-subjects effects table. The criterion for statistical significance was *p* < 0.05. Fisher's Least Significant Difference procedure was used for all post hoc tests. Normality of all measures was assessed by inspecting normal probability plots.

## Results

### Pretraining and TUNL training

One DBA/2J mouse (n = 1) failed to acquire the TUNL task. Consequently, the analyses reported below were performed using the following sample: C57BL/6J mice (n = 22) and DBA/2J mice (n = 20). During pretraining, mice learned to collect a reward, nosepoke the touchscreen, initiate the choice phase, and initiate the sample phase (Table S1). C57BL/6J mice completed the four pretraining stages in fewer sessions than DBA/2J mice [F (1, 40) = 5.30, *p* < 0.05] (Figure S1). Following pretraining and prior to TUNL testing, mice learned the TUNL assay during nine training stages (Table S2). ANOVA revealed that the number of sessions required for C57BL/6J mice and DBA/2J mice to complete the training stages did not differ significantly (Figure S2a). Strain did not affect the number of total trials, omitted trials, or correction trials on the training stages (Figure S2b,c,d).

### TUNL testing: TUNL performance was significantly influenced by strain, delay, horizontal distance, and vertical distance

TUNL performance of C57BL/6J mice was significantly better than performance of DBA/2J mice (Fig. 2a) [F (1, 40) = 9.83, *p* < 0.01]. Increasing delay significantly impaired TUNL performance (Fig. 2b) [F (3, 38) = 32.03, *p* < 0.001]. Increasing horizontal distance significantly *facilitated* TUNL performance (Fig. 2c,d) [F (2, 39) = 58.33, *p* < 0.001]. In contrast, increasing vertical distance significantly *impaired* TUNL performance (Fig. 2e,f) [F (1, 40) = 42.16, *p* < 0.001]. As described below, the effects of delay and horizontal distance, but not vertical distance, on TUNL performance were significantly influenced by strain.

### TUNL testing: mouse strain significantly influenced the effect of delay and horizontal distance, but not vertical distance, on TUNL performance

**Figure 3:**
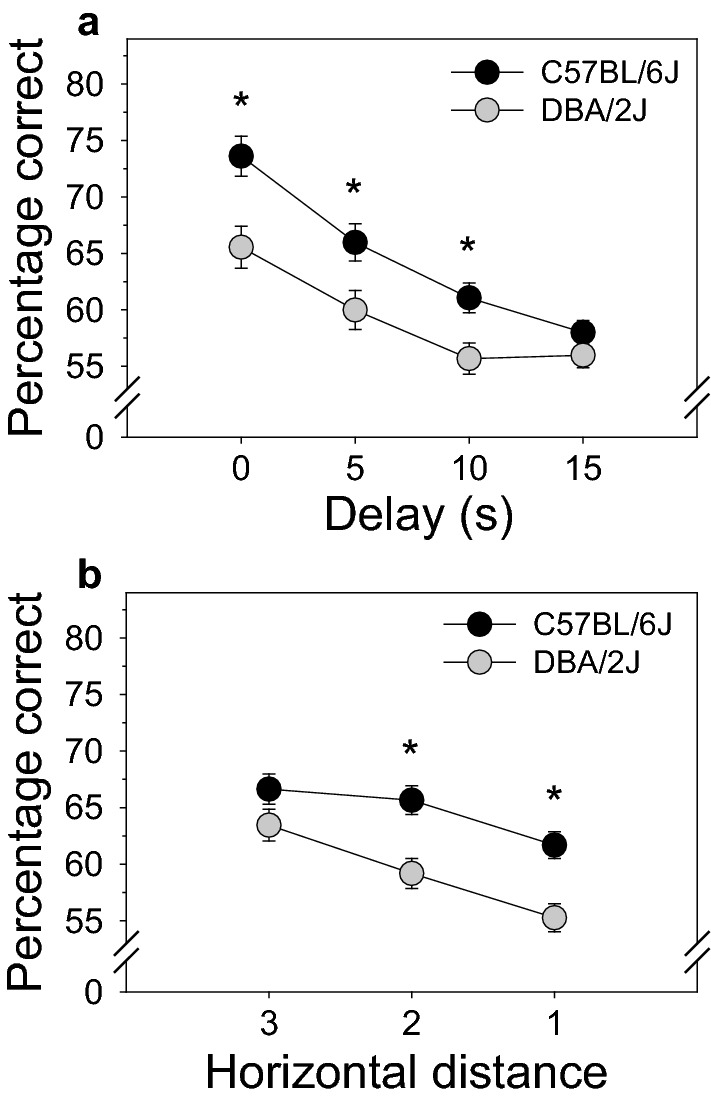
Mouse strain significantly influenced the effect of delay and horizontal distance, but not vertical distance, on TUNL performance in C57BL/6J and DBA/2J mice (**a**) Percentage correct on the 0, 5, and 10 s delay was significantly higher in C57BL/6J mice than in DBA/2J mice. Performance of C57BL/6J mice and DBA/2J mice did not differ significantly on the 15 s delay. The largest difference between C57BL/6J and DBA/2J mice was observed on the 0 s delay during which working memory would be expected to have a negligible impact on performance. **(b)** Performance of C57BL/6J mice and DBA/2J mice did not differ on the easiest discrimination during which the two choice-phase stimuli were displayed at the widest distance. In contrast, performance of DBA/2J mice was significantly impaired relative to C57BL/6J mice on more difficult discriminations during which stimuli were displayed at narrower distances. The two-way interactions illustrated here are fully decomposed in Fig. 4. Error bars represent SEM. * *p* < .05.

Mouse strain significantly influenced the effect of delay on TUNL performance [F (3, 38) = 3.17, *p* < 0.05]. Post hoc tests revealed that percentage correct on the 0, 5, and 10 s delay was significantly higher in C57BL/6J mice than in DBA/2J mice (Fig. 3a). Performance of C57BL/6J mice and DBA/2J mice did not differ significantly on the 15 s delay. The largest difference between C57BL/6J and DBA/2J mice was observed on the 0 s delay during which working memory would be expected to have a negligible impact on performance.

Mouse strain significantly influenced the effect of horizontal distance on TUNL performance [F (2, 39) = 3.65, *p* < 0.05]. Post hoc tests revealed that performance of C57BL/6J mice and DBA/2J mice did not differ on the easiest discrimination during which the two choice-phase stimuli were displayed at the widest distance (Fig. 3b). In contrast, performance of DBA/2J mice was significantly impaired relative to C57BL/6J mice on more difficult discriminations during which stimuli were displayed at narrower distances.

**Figure 4:**
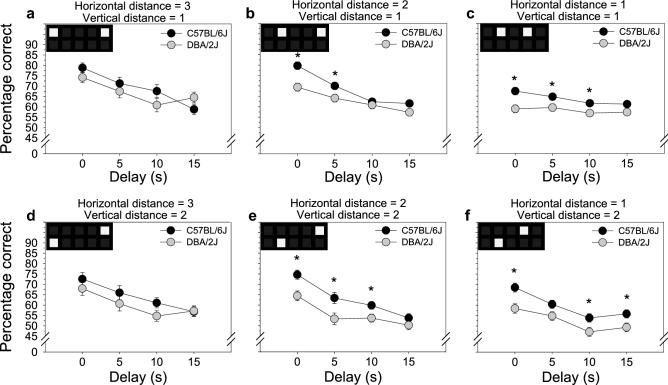
Pattern separation, but not working memory, is significantly impaired in DBA/2J mice relative to C57BL/6J mice^1^. (**a**,**d**) TUNL performance of C57BL/6J mice and DBA/2J mice was equivalent at all delays when choice-phase stimuli were presented at the widest horizontal distance. **(b,c,e,f)** In contrast, when choice-phase stimuli were presented at an intermediate horizontal distance and narrow horizontal distance, performance of DBA/2J mice was significantly impaired relative to C57BL/6J mice. This performance impairment was observed at multiple delays, most consistently at the 0 s delay when pattern separation but not working memory would be expected to account for performance variation. This horizontal separation deficit was observed irrespective of vertical distance between choice-phase stimuli (compare top row panels vs bottom row panels). Error bars represent SEM. * *p* < .05. ^1^Stimuli in the top left of each panel represent one example of a choice-phase stimuli configuration within the horizontal and vertical distance category combination. There were multiple possible stimuli configurations within each category combination, and all configurations were used in the TUNL experiment.

When these two-way interactions are decomposed (Fig. 4), it reveals that performance of C57BL/6J mice and DBA/2J mice was equivalent at all delays when choice-phase stimuli were presented at the widest horizontal distance (Fig. 4a,d). In contrast, when choice-phase stimuli were presented at an intermediate horizontal distance (Fig. 4b,e) and narrow horizontal distance (Fig. 4c,f), performance of DBA/2J mice was significantly impaired relative to C57BL/6J mice. This performance impairment was observed at multiple delays, most consistently at the 0 s delay when pattern separation but not working memory would be expected to account for performance variation. This horizontal separation deficit was observed irrespective of vertical distance between choice-phase stimuli (Fig. 4a vs. d, b vs. e, c vs. f).

## Discussion

### Summary

Using the TUNL touchscreen operant conditioning assay, we assessed the effects of mouse strain (C57BL/6J, DBA/2J), delay between the sample and choice phase (0, 5, 10, 15 s), horizontal distance between choice-phase stimuli (1, 2, 3), vertical distance between choice-phase stimuli (1, 2), and interactions among these variables on TUNL performance. We identified statistically significant main effects of all four independent variables (Fig. 2): C57BL/6J mice exhibited superior TUNL performance relative to DBA/2J mice; increasing delay between the sample and choice phase impaired TUNL performance; increasing horizontal distance facilitated TUNL performance; and increasing vertical distance impaired TUNL performance. In addition to these main effects, we observed a statistically significant interaction of strain and delay as well as a statistically significant interaction of strain and horizontal distance (Fig. 3). Decomposition of these effects (Fig. 4) revealed that TUNL performance of C57BL/6J mice and DBA/2J mice was equivalent at all delays when choice-phase stimuli were presented at the widest horizontal distance. In contrast, when choice-phase stimuli were presented at narrower distances (i.e., relatively more difficult discriminations), performance of DBA/2J mice was significantly impaired relative to C57BL/6J mice. This performance impairment was observed at multiple delays, most consistently at the 0 s delay when pattern separation but not working memory would be expected to account for performance variation. Collectively, these data reveal a pattern separation impairment, but not working memory impairment, in DBA/2J mice relative to C57BL/6J mice.

### Effects of task parameters on TUNL performance in mice: delay, horizontal distance, and vertical distance

In the present study, we observed both expected and unexpected effects of task parameters on TUNL performance. The effects of delay (Fig. 2b) and horizontal distance (Fig. 2c,d) were as expected and were consistent with findings from the initial TUNL studies^22^. Specifically, performance declined as duration of the delay increased, and performance improved as horizontal distance increased. In contrast, the effect of vertical distance on performance was not as expected. Specifically, during the choice phase, performance of mice was significantly better when the sample stimulus and novel stimulus were presented on the same row (closer together) rather than on different rows (farther apart) (Fig. 2e,f). This observation contrasts with the general observation from the initial TUNL studies (performed using rats) that pattern separation is easiest when stimuli are farther apart. One possible explanation for the counterintuitive finding in the present study is that mice were biased to respond to either the top row or bottom row. When stimuli were on the same row, a top or bottom bias could not have affected performance. In contrast, when stimuli were on different rows, a bias to respond to the top or bottom row would have impaired performance. In future studies, this issue could be addressed by excluding vertical distance as a variable (i.e., using only a single row of stimuli). To our knowledge, the distinct effects of horizontal and vertical distance on TUNL performance have never been dissociated. Rather, distance between stimuli has been considered a single variable that has been manipulated by increasing both vertical and horizontal distance^22^. Findings from the present study suggest that, at least in mice, horizontal distance and vertical distance should be treated as distinct independent variables on the TUNL assay.

### Pattern separation, but not working memory, is significantly impaired in DBA/2J mice relative to C57BL/6J mice

In the present study, TUNL performance of C57BL/6J mice and DBA/2J mice was equivalent at all delays when choice-phase stimuli were presented at the widest horizontal distance (Fig. 4a,d). This indicates that on relatively easy discriminations (i.e., Fig. 2c,d), pattern separation is equivalent in C57BL/6J mice and DBA/2J mice. However, on more difficult discriminations during which choice-phase stimuli were presented relatively close together, performance of DBA/2J mice was impaired relative to C57BL/6J mice (Fig. 4b,c,e,f). These data indicate that the effect of horizontal distance on performance was significantly dependent on strain. It is notable that this impairment was observed irrespective of vertical distance (compare top and bottom rows of Fig. 4), and that no significant interaction was observed between strain and vertical distance. Regarding working memory, it is notable that the most robust strain difference on difficult discriminations (Fig. 4b,c,e,f) was observed at the 0 s delay on which working memory would not be expected to influence performance. Collectively, these data indicate the existence of a deficit in pattern separation, but not working memory, in DBA/2J mice relative to C57BL/6J mice.

It should be noted that DBA/2J mice begin to develop glaucoma at six months of age^40^. The mice in the present study completed testing before six months of age. Thus, the loss of vision in DBA/2J mice is unlikely to have affected TUNL performance in the present study. However, low-level differences in the visual system or in the processing of visual information could account for strain differences observed in the present study. Moreover, differences between mice and rats in these systems may explain species differences in TUNL performance, including effects on vertical distance discrimination. Integration of in vivo techniques (e.g., calcium imaging, electrophysiology) with the TUNL assay could confirm this hypothesis and enable dissection of identified strain dependent and species dependent mechanisms.

Because the present study was restricted to male mice, the inclusion of sex as a factor in future TUNL studies may enable identification of strain differences in working memory. This is supported by findings that sex hormones influence working memory and other cognitive functions^41–43^. Moreover, sex hormones have been shown to influence performance on the TUNL assay^44^.

To our knowledge, this is the first study to quantify pattern separation in different mouse or rat strains. Therefore, these data reveal for the first time that pattern separation is a heritable phenotype in mice, and that this cognitive ability can be quantified using the TUNL assay. Regarding working memory, data from the present study are consistent with past studies in which equivalent working memory between C57BL/6J and DBA/2J mice was observed on the lever-based delayed matching to position^45^ and delayed non-matching to position tasks^46,47^. Despite agreement among the present study and past studies, it is possible that working memory differences between C57BL/6J and DBA/2J mice may ultimately be identified. For example, in the present study, the inter-trial interval (ITI) was held constant at five seconds. In this regard, prior studies of working memory indicate that short ITIs impair performance due to proactive interference^48,49^. Thus, experimentally manipulating variables such as ITI duration may reveal strain dependent effects.

### Discovery of genetic mechanisms driving pattern separation ability using the BXD recombinant inbred mouse panel

Data presented here reveal that pattern separation is a heritable phenotype in mice that is driven by, at least in part, genetic differences that exist in the C57BL/6J and DBA/2J inbred strains. These two strains are the founders of the BXD recombinant inbred panel^21^. The genome of each of the ~ 140 BXD strains consists of a unique and random combination of C57BL/6J and DBA/2J alleles. Consequently, a systems genetics approach using the BXD panel^24–31^ would enable discovery of the genetic mechanisms underlying the heritable variation in pattern separation observed in this study. Although working memory of the C57BL/6J and DBA/2J founder strains appears to be largely equivalent when quantified using food-reinforced operant conditioning tasks^45–47^ and this study, working memory of BXD strains is more likely to be divergent relative to founders because of transgressive segregation^50,51^. In future studies, this hypothesis could be tested by quantifying working memory using the TUNL assay in a randomly selected subset of BXD strains.

## Supplementary Information

Supplementary Information.
