# Supplementary material for: Working memory and pattern separation in founder strains of the BXD recombinant inbred mouse panel
Source: Sci Rep. 2022 Jan 7;12:69. doi: 10.1038/s41598-021-03850-3 (PMC8741792; doi:10.1038/s41598-021-03850-3)
Supplement: Supplementary file 1 — Supplementary Information. [file 41598_2021_3850_MOESM1_ESM.pdf]

Working memory and pattern separation in founder strains of the BXD recombinant inbred mouse panel

Price E. Dickson[*1, 3] and Guy Mittleman[2, 3]

1. Department of Biomedical Sciences
   Joan C. Edwards School of Medicine
   Marshall University
   1700 3rd Ave.
   Huntington, WV 25703

2. Department of Psychological Science
   North Quad (NQ), room 104
   Ball State University
   Muncie, IN 47306

3. Department of Psychology
   University of Memphis
   400 Innovation Drive
   Memphis, TN 38111

* Corresponding author
Price E. Dickson, Ph.D.
price.dickson@marshall.edu

Keywords:
TUNL
C57BL/6J
DBA/2J
systems genetics
systems biology
short term memory
touchscreen
operant
executive function

Table S1. TUNL pretraining

Stage[1]	Sequence of behaviors required to complete a trial
1	· collect reward
2	· nosepoke stimulus on touchscreen[2]<br>· collect reward
3	· initiate sample phase with nosepoke to food receptacle[3]<br>· nosepoke stimulus on touchscreen<br>· collect reward
4	· initiate sample phase with nosepoke to food receptacle<br>· nosepoke stimulus on touchscreen<br>· initiate choice phase with nosepoke to food receptacle<br>· nosepoke stimulus on touchscreen<br>· collect reward

[1.] On each TUNL pretraining stage, mice were required to complete at least 20 trials during the 60-minute session before advancing to the next stage.

[2.] During pretraining, only a single stimulus was presented during the sample phase and choice phase. The visual stimulus used during pretraining was identical to the visual stimulus used during the TUNL assay and was presented randomly in one of the 10 positions in the 2 x 5 matrix. The position of the stimulus was randomized independently for the sample phase and choice phase.

[3.] The stimulus light located above the food receptacle was illuminated to signal that the sample phase or choice phase could be initiated.

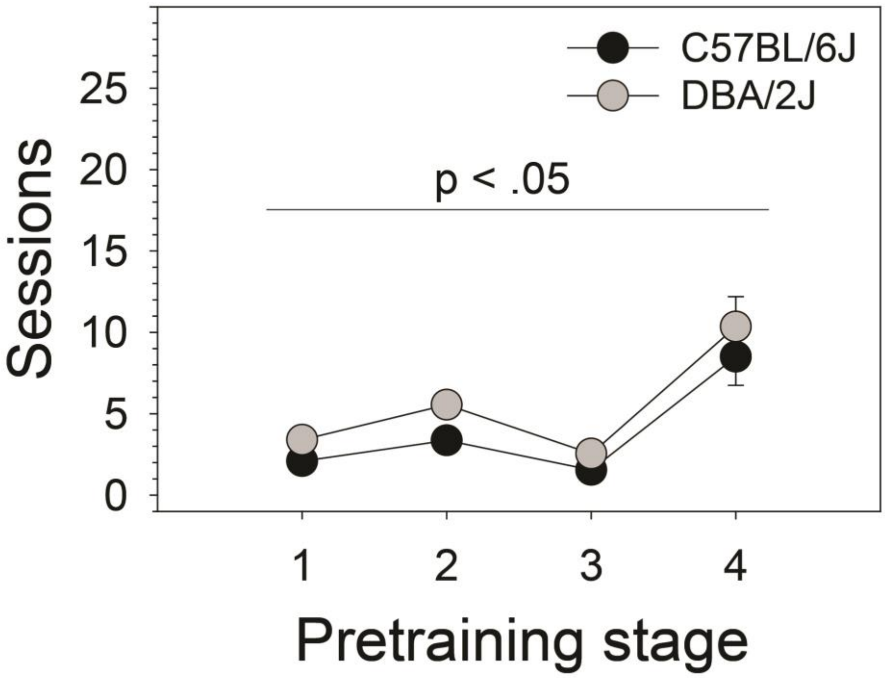

Figure S1. Pretraining stages. During pretraining, mice learned to collect a reward, nosepoke the touchscreen, initiate the choice phase, and initiate the sample phase (Table S1). C57BL/6J mice completed the four pretraining stages in fewer sessions than DBA/2J mice [F (1, 40) = 5.30, p < .05]. There was a significant effect of pretraining stage [F (3, 38) = 12.89, p < .001] indicating that some stages were completed more rapidly than others. However, this effect was unrelated to the effect of strain as indicated by the absence of a pretraining stage x strain interaction.

Table S2. TUNL training

Stage[1]	Delay (s)				Horizontal distance category[2]		
	A	B	C	D	3	2	1
1	0	-	-	-	Yes	-	-
2	0	-	-	-	Yes	Yes	-
3	0	-	-	-	Yes	Yes	Yes
4	0	2.5	-	-	Yes	Yes	Yes
5	0	2.5	5	-	Yes	Yes	Yes
6	0	2.5	5	7.5	Yes	Yes	Yes
7	0	5	7.5	10	Yes	Yes	Yes
8	0	5	10	12.5	Yes	Yes	Yes
9[3]	0	5	10	15	Yes	Yes	Yes

1. On each TUNL training stage, mice were required to reach a criterion of 80% correct on trials that used a zero second delay and the widest horizontal distance between choice-phase stimuli (i.e., the least challenging discrimination) before moving to the next TUNL training stage. Once mice had reached that criterion on each of the nine training stages, TUNL testing began. The session was terminated when mice completed 64 trials (excluding omitted trials) or when 60 minutes had elapsed, whichever occurred first.

2. Both categories of vertical distance were used at all training stages.

3. Stage 9 variable values were identical to those used during TUNL testing. Mice were required to meet criterion at training stage 9 prior to advancing to the TUNL testing stage.

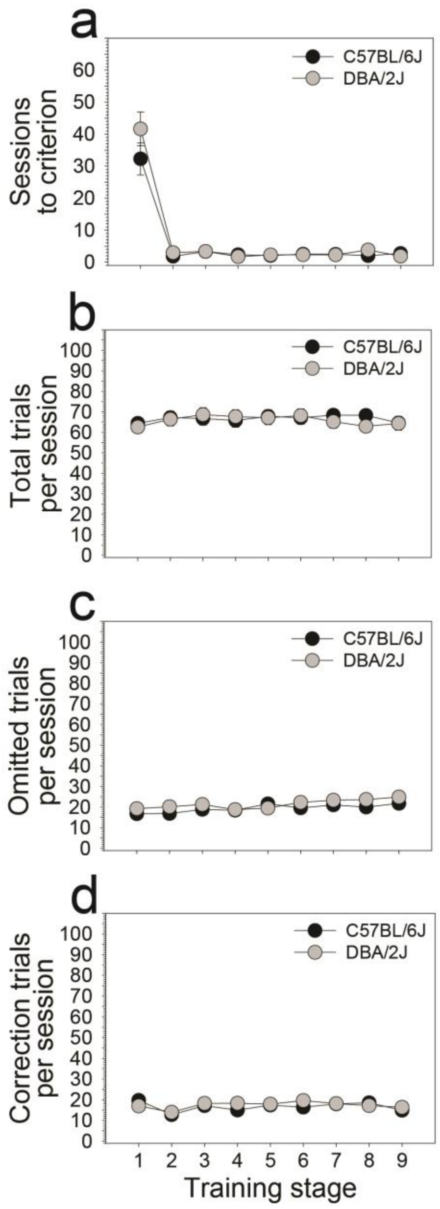

Figure S2. Training stages. Following pretraining and prior to TUNL testing, mice learned the TUNL assay during nine training stages (Table S2). **(a)** ANOVA revealed that the number of sessions required for C57BL/6J mice and DBA/2J mice to complete the training stages did not differ significantly. There was a significant main effect of training stage on sessions [$F(8, 33) = 17.84$, $p < .001$] which was strongly driven by the relatively high number of sessions on stage one; this phenomenon reflects the difficulty of learning to discriminate between the sample and novel stimuli which occurred on stage one. However, this effect was unrelated to the effect of strain as indicated by the absence of a training stage x strain interaction. **(b, c, d)** Strain did not affect the number of total trials, omitted trials, or correction trials on the training stages.
